# Heartless Rulez! Mechanical objectivity, empathic understanding, and the permissibility of AI

**DOI:** 10.3389/fsoc.2025.1604709

**Published:** 2025-07-08

**Authors:** Patrick Schenk

**Affiliations:** University of Lucerne, Lucerne, Switzerland

**Keywords:** artificial intelligence, morality, objectivity, empathy, social and technological orientations, survey experiment

## Abstract

Sociologists have generally stressed AI’s capacity to make processes mechanically objective as a major justification for its use in modern societies. Psychologists, in contrast, have emphasized AI’s lack of empathic understanding as a major barrier for its moral acceptance. From the perspective of mechanical objectivity, a process is considered legitimate and fair if it maximizes consistency through the impersonal application of rules. Coming from empathic understanding, a purely mechanically objective process is inflexible, deterministic, and heartless. Mechanical objectivity and empathic understanding are thus in tension. This paper empirically analyzes the impact of mechanical objectivity, empathic understanding, and their interplay with an individual’s general orientations for permissibility judgments on the use of AI as an adjudicating entity in criminal courts. In a survey experiment with 793 students in Switzerland, I find that both concepts causally impact permissibility. Yet, social orientation significantly moderates the effect of empathic understanding. Socially oriented individuals are thus particularly skeptical of AI as an adjudicating entity because of its deficit to emphasize with others. The study demonstrates the importance of theorizing the interplay between cultural concepts and internalized orientations to explain the impact of normative ideals on the acceptance of AI, bringing sociological and psychological research into conversation.

## 1 Introduction

Technologies are morally embedded. Their development, implementation, and use need to be perceived as legitimate, justified, or permissible by major stakeholders and the general population (Chan and Lo, 2025). Artificial intelligence (AI) is no exception. According to sociological research, a major justification to use AI lies in its alleged capacity to make processes more objective, consistent, controllable, and accountable (Brayne and Christin, 2021; Burrell and Fourcade, 2021; Geser, 1989; Lavanchy et al., 2023; Petre, 2018; Rosen et al., 2021; Sartori and Bocca, 2023; Zajko, 2021). According to psychological research, a major precondition for the acceptance of using AI systems for moral decisions lies in the attribution of mind to artificial agents (Bigman and Gray, 2018; Gamez et al., 2020; Shank and DeSanti, 2018; Shank et al., 2021; Waytz et al., 2010). Yet, these two perspectives are in tension. Not only do they provide different explanations for the permissibility of AI. They describe conflicting justifications for its use.

This is a conflict between ideals of mechanical objectivity and empathic understanding. In a nutshell, mechanical objectivity rests on the impersonal application of rules (Burrell and Fourcade, 2021; Daston and Galison, 1992; Porter, 1992). Empathic understanding means the capacity to share the emotional and motivational states of other individuals (Bierhoff, 2002; Bigman and Gray, 2018; Riek et al., 2009). The former is best realized by machines and in the absence of mind, while the latter presupposes experiential mind. From the perspective of mechanical objectivity, a process is considered legitimate and fair if it maximizes consistency (Luhmann, 1978). From the perspective of empathic understanding, a process is considered permissible if it enables a comprehensive interpretation of unique circumstances (Lavanchy et al., 2023; Nagtegaal, 2021). Coming from mechanical objectivity, a process solely relying on empathic understanding is prone to arbitrariness and special treatment. Coming from empathic understanding, a purely mechanically objective process is inflexible, deterministic, and heartless.

The conflict between mechanical objectivity and empathic understanding is omnipresent in AI discourses and practices (Clark and Gevorkyan, 2020; Hoffman et al., 2022; Petre, 2018; Rosen et al., 2021). Yet, it is especially obvious in legaltech (Soukupová, 2021). Nowadays, technologies of AI are being used in nearly all areas of the criminal justice system in many places of the world, form the US to China to Switzerland, from the provision of legal documents to predictive policing to risk assessment tools (Stevenson and Doleac, 2019; Xu et al., 2022; Završnik, 2021). In criminal courts, these tools play a more and more important role in sentencing procedures, such as setting bail amounts, determining prison sentences, prison placement, or parole decisions (Brayne and Christin, 2021; Hayward and Maas, 2021). The software COMPAS is a particularly infamous example (Xu et al., 2022).

Proponents and skeptics alike refer to notions of mechanical objectivity or empathic understanding to justify or criticize the use of AI systems in criminal courts. On the one hand, in line with the ideal of mechanical objectivity, systems of AI could ensure that everybody is equally treated under the law by the impersonal application of standardized rules. On the other, in line with the ideal of empathic understanding, the unique circumstances of individual cases essentially include the motivations and feelings of the involved parties (cf. Machura, 2017; Thibaut and Walker, 1975; Xu et al., 2022). Of course, these normative justifications do not necessarily mirror the actual functioning of technologies. For example, the software COMPAS has been accused of being biased against Black people (Farayola et al., 2023). Nevertheless, mechanical objectivity and empathic understanding provide powerful normative frames for assessing the permissibility of AI. Reflecting on automated justice, Fabian (2020, p. 6) writes: “At this point of our evolution […], we must not forget that judging requires not only knowledge of the law […], but also the empathetic ability to understand the emotions and motivations underlying human behaviour.”

In general, AI can be defined as an artifact which is “capable of displaying […] behaviors that we consider to be intelligent” (Arkoudas and Bringsjord, 2014, p. 34). The conflict between mechanical objectivity and empathic understanding is particularly salient here because people tend to locate AI between mindless tools and fully-fledged human beings (Gray et al., 2007; Shank et al., 2021). It simultaneously embodies both capacities to various extents. Yet, the tension between mechanical objectivity and empathic understanding is by far not limited to AI. It has a long history in western culture, going back to enlightenment and romanticism, showing up in debates on science or bureaucracy, for example (Daston and Galison, 1992; Petre, 2018; Porter, 1992; Weber, 2019). The tension between the application of impersonal rules and individual circumstances surfaces in a myriad of everyday situations. Just think about getting a parking ticket for being 3 min late because you helped a good friend finding her keys. Mechanical objectivity and empathic understanding are cultural concepts. They are part of a historically evolved stock of shared knowledge and understandings. People use these concepts to make sense of various situations and their moral implications (Abend, 2014). They might also put them to work for AI (Beer, 2016; Mays et al., 2022).

In this paper, I empirically analyze the causal impacts of mechanical objectivity and empathic understanding on the permissibility of using AI as an adjudicating entity in courts. How do people balance these conflicting ideals and react to this tension? This is the first research question. Furthermore, I argue that the impact of mechanical objectivity and empathic understanding varies in theoretically predictable ways between individuals depending on their general orientation towards technology and sociality. Technological orientations denote a need for the interaction with new technologies, while social orientations denote the need for the interaction with others and social solidarity (Hashinaga et al., 2023; Horstmann and Krämer, 2019; Ito, 1994). This is the second research question. I study these questions by a survey experiment conducted with 793 university students in Switzerland. I thus focus on non-experts to understand the perception of AI among a group that is of special interest for the implementation and regulation of artificial intelligence. Individuals with higher education are statistically more likely to attain positions of power, to become entrepreneurs, to vote, and to be carriers of social activism (Dahlum and Wig, 2021). As an individualized country with high trust in the juridical system but limited prior experience with AI and human-robot interactions, Switzerland provides a context in which the tension between mechanical objectivity and empathic understanding might be especially pronounced (Hofstede and McCrae, 2004; Latzer and Festic, 2024; OECD, 2024).

The impact of normative criteria on the acceptance of AI among non-experts is still underresearched (Kieslich et al., 2022; Mantello et al., 2023; Mays et al., 2022; Shin, 2022; Yigitcanlar et al., 2022). To the best of my knowledge, this is the first contribution juxtaposing the opposing goals of mechanical objectivity and empathic understanding, studying their trade-offs, and theorizing their interplay with an individual’s general orientation. Surveying ethical challenges is essential to explain the reception of AI on the ground. Ethnographic and quantitative research on the implementation of AI algorithms in courts have shown that their use is often discontinued by judges due to a lack of trust (Brayne and Christin, 2021; Stevenson and Doleac, 2019). Studies have also found that algorithms are generally perceived as less procedurally fair than humans (Lavanchy et al., 2023). Especially when AI takes over positions of power, pertinent questions of moral and political legitimacy arise (Danaher, 2016; Nagtegaal, 2021; Xu et al., 2022). Scholars have pointed out various normative issues of AI, ranging from transparency to the protection of human rights and civil liberties such as non-discrimination, freedom, equality, and the right for a fair trial (Chan and Lo, 2025). Indeed, the AI Act by the European Union—the first legal framework on AI—classifies the use of AI in the administration of justice and democratic processes as a high-risk case (European Union, 2024). Research on AI in powerful positions is thus of great urgency (Mantello et al., 2023). From a theoretical perspective, the present research contributes to the sociologies and psychologies of morality and AI by providing new evidence for concurrent explanations of AI’s permissibility (Bigman and Gray, 2018; Hitlin et al., 2023; Hoffman et al., 2022).

In a first step, I explain the concepts of mechanical objectivity and empathic understanding in more detail. I define and characterize these concepts, locate them in AI discourse, and demonstrate their connection to judgements of permissibility. In the second step, I explain why technological and social orientations should moderate the impact of mechanical objectivity and empathic understanding. I derive four hypotheses from the theoretical discussion. In the third section, I present the data and methods, before presenting the results in the succeeding section. The final section summarizes the main findings, acknowledges limitations, shows avenues for future research, and discusses the practical and theoretical implications.

## 2 Theory

### 2.1 Mechanical objectivity

The literature is anything but short of references to objectivity as a major advantage of using AI. In narratives and discourse, AI is often framed as being more objective, neutral, impartial, unbiased and reliable compared to their human counterparts, providing a technological solution to moral problems, such as discrimination and injustice (Burrell and Fourcade, 2021; Lavanchy et al., 2023; Sartori and Bocca, 2023). In an ethnographic study by Brayne and Christin (2021), for example, judges and police officers justified the use of predictive algorithms as a means to mitigate bias, improving the reliability of judgments, and objectivizing the decision process (see Figueroa-Armijos et al., 2023 for the field of personal recruitment). However, as these examples already hint at, the modern notion of objectivity fuses heterogenous but logically independent components (Risjord, 2014). In this paper, I focus on “mechanical objectivity.”

In essence, mechanical objectivity refers to impersonality (Daston and Galison, 1992; Porter, 1992; Risjord, 2014). It is best contrasted to subjectivity. Mechanical objectivity demands that an outcome does not involve any personal judgment, implying interpretation and selectivity. Instead, outcomes should be produced according to standardized rules. These rules allow the detachment of a process from individual volition and discretion. In this sense, mechanical objectivity seeks to eliminate human intervention. Operationally, it can be defined as the degree of consensus among a group of observers. Modern law is a formidable example of mechanical objectivity (Weber, 2019). Judges should refer to a systematic body of rules and precedents instead of simply following their personal preferences and understandings. Other prominent examples of mechanical objectivity are photographical reproduction in science (Daston and Galison, 1992), modern accounting methods (Porter, 1992), or algorithmic metrics in journalism (Petre, 2018). As these examples suggest, a complete absence of human intervention and absolute impersonality are the ideals of mechanical objectivity, never being fully realized in reality (Porter, 1992).

From the perspective of mechanical objectivity, a process or outcome is considered legitimate, fair, acceptable, or right when it guarantees a maximum degree of consistency enabled by the impersonal application of standardized rules. Consistency stands in contrast to arbitrariness or “special treatment” (Nagtegaal, 2021). As the sociologist Luhmann (1978) has pointed out, rules legitimize procedures in modern societies. Accordingly, the literature on procedural fairness has identified consistency as one of the major determinants of fairness perceptions in the criminal justice system (Machura, 2017; Thibaut and Walker, 1975). Likewise, Farayola et al. (2023) underscore consistency as an essential requirement for trustworthy AI in the context of risk assessment tools. Offenders with similar charges should receive the same risk score, ceteris paribus. Xu et al. (2022, p. 1604) provide an extensive explanation of how AI could “solve the problem of similar cases be decided similarly” in the context of sentencing, referring to standardized procedures for information processing and forming judgments by rational syllogisms. More generally, mechanical objectivity is a constitutive ingredient for legitimate power in modern societies (Burrell and Fourcade, 2021; Geser, 1989; Nagtegaal, 2021; Porter, 1992). As Weber (2019) described, the legitimacy of modern rational rule, exemplified by bureaucratic administration, derives from the application of universal and binding rules without regard for persons, giving everyone the same impartial consideration, in contrast to making decisions based on personal sympathy and favor.

Mechanical objectivity is conceptually distinct from bias or reliability (Risjord, 2014). Bias refers to the systematic discrimination of people based on group membership (Zajko, 2021). Reliability refers to the ability to repeatedly produce a correct result (Dietvorst et al., 2015). Imagine an algorithm predicting the risk of recidivism. It neatly follows a limited set of standardized rules, producing the same output with the same input, hence being fully mechanically objective (Stevenson and Doleac, 2019). Yet, the algorithm could still discriminate against People of Color or fail to predict recidivism (see for example Završnik, 2021). Thus, a process could be fully mechanically objective, while being biased and unreliable. A mechanically objective process might even reproduce and perpetuate bias (Espeland and Yung, 2019; Zajko, 2021). It might also be unresponsive to innovations improving reliability (Završnik, 2021). Scholars have therefore criticized the conservative tendency of mechanical objectivity (Zajko, 2021). Hence, a process might be fully objective from the perspective of mechanical objectivity, while utterly failing to be so given different conceptions of objectivity, such as bias or reliability (Daston and Galison, 1992).

There is a strong elective affinity between mechanical objectivity and algorithms (or machines more generally). For human agents, following standardized rules takes a large deal of self-discipline. Machines, in contrast, are inherently rule-following. By definition, algorithmization consists of translating a process into a sequence of simple and clear commands, i.e., rules (Rammert, 2016). Moreover, mechanical objectivity does not presuppose mind or freedom of will (Geser, 1989). Quite the contrary. The virtue of machines lies exactly in the freedom from will, the absence of mind and subjectivity, unburdening a process from the temptation to deviate from standardized rules (Daston and Galison, 1992). Indeed, the more dehumanized a process, the higher the amount of social control, the more blindly agents follow rules, the more perfectly developed is mechanical objectivity (Burrell and Fourcade, 2021; Geser, 1989). Given this perspective, algorithms are the paragon of certain epistemic virtue and moral values (Beer, 2016). “What the human observer could achieve only by iron self-discipline, the machine achieved willy-nilly” (Daston and Galison, 1992, p. 120). Algorithms are seen as the embodiment of computational fairness, the application of rules in a consistent, controllable, and impersonal way (Burrell and Fourcade, 2021; Shulner-Tal et al., 2023). By extension, this implies an affinity of artificial intelligence with judicial justice, grounded on consistency and logical reasoning, as Xu et al. (2022) contend. Finally, the imaginary of machines as a hallmark of mechanical objectivity is further strengthened by quantification (Porter, 1992). Quantification is enabled by standardization, making complex reasoning processes about qualitative differences amenable to computation. Representing a process in numbers, as it is the case with AI algorithms, commands a specific kind of authority in modern societies (Espeland and Yung, 2019). Quantification solidifies the impression that a process is impersonal and hence mechanically objective.

### 2.2 Empathic understanding

The literature is not short of critics of mechanical objectivity either. An especially prominent line of criticisms juxtaposes mechanical objectivity to empathic understanding as an essentially human quality (Burrell and Fourcade, 2021; Clark and Gevorkyan, 2020; Kieslich et al., 2022; Porter, 1992; Rosen et al., 2021; Završnik, 2021). From this perspective, human judgements involve intuition, personal experience, compassion, sympathy, affect, and feelings, in contrast to simply following impersonal rules (Farayola et al., 2023; Geser, 1989). Judgments do not solely rely on measurable and quantifiable characteristics (Nagtegaal, 2021; Porter, 1992). These processes are uncodifiable, especially when it comes to the moral domain (Gamez et al., 2020). Standardization leads to deterministic, inflexible, and right-out “dumb” behavior (Brayne and Christin, 2021). Blindly following rules might even produce harmful outcomes without an agent’s malicious intent, obscuring responsibilities, famously described by Hannah Arendt (2022) as the banality of evil. Fairness is not just about consistency, then, but about solidarity, community, and the particularity of individual circumstances (Rosen et al., 2021). No single set of rules is able to account for the diversity of experiences and life circumstances of people within intersecting dimensions of social inequality and local contexts (Collins et al., 2021). This is also true for the judicial system, as Xu et al. (2022) or Fabian (2020) discuss, pointing out the pitfalls when judicial processes are reduced to impersonalized mechanical operations. In short, for “cold-blooded machines […], humanness is less […] a feature than a bug” (Završnik, 2021, p. 13), reducing qualitative judgments to “the kind of language that even a thing as stupid as a computer can use,” (Porter, 1992, p. 644).

Given these accounts, the concept of empathic understanding refers to the capacity to share another person’s intentional emotional and motivational states (Bierhoff, 2002; Riek et al., 2009; Tronto, 1998). It demands the ability to put oneself in someone’s shoes (Nallur and Finlay, 2023), even when disagreeing with the other person (Kleinrichert, 2024). This does not only pertain to a cognitive but also to an emotional level, to feel pleasure or pain on the behalf of others, enabling a true understanding of their motivations (Bigman and Gray, 2018). The ability for empathic concern is therefore tightly connected to experiential mind. In contrast to agentic mind, which denotes the ability to have intentions, experiential mind refers to the ability to sense and feel (Shank et al., 2021). It includes basic biological states, such as a hunger or pain, but also more complex emotions, such as pride or joy (Lee et al., 2021). Only if an agent has affective capacities, they are able to truly share, and not merely simulate, another person’s internal states (Arkoudas and Bringsjord, 2014).

According to empirical research, respondents attribute less experiential mind to AI than humans (Gray et al., 2007; Shank and DeSanti, 2018). Respondents are also unwilling to use intelligent machines as an emotional replacement for human partners (Korn et al., 2021). However, the tendency to attribute humanlike mental capacities to nonhuman entities varies individually and situationally (Waytz et al., 2010). For example, in an experimental study by Lee et al. (2021), respondents used more emotionally valanced messages when an AI has been framed to feel emotions and to have a heart. At least for some people then, AI is situated between completely mindless tools and fully minded humans (Shank et al., 2021).

From the perspective of empathic understanding, a process or outcome is considered legitimate, fair, acceptable or right when it takes the emotional and motivational states of the involved individuals into account. At least three arguments have been made for this.

First, experiential mind has been linked to moral agency. For some, moral judgements are more strongly grounded in spontaneous emotional reactions, feeding into moral intuitions, than in universal moral rules or the calculation of utilities (Audi, 2022; Bigman and Gray, 2018; Lavanchy et al., 2023). Prominently, the political theorist Joan Tronto (1998, 2020) argues for an ethics of care. According to her, morality is not grounded in abstract principles but enacted in the practice of caring for others and oneself. Caring is underpinned by the sympathetic appreciation of emotions and calls attention to the interdependence of human beings (Jesenková, 2022). For Tronto, caring is part of the human experience—a species activity that makes us human. It should serve as a fundamental value of social life, be it in close relationships, political systems, or bureaucratic institutions (Tronto, 1998). This line of reasoning resembles current interventions in AI ethics. As Nallur and Finlay (2023) have argued, the ethics of AI has focused too narrowly on big normative ideas, such as justice or bias, while neglecting the social and relational aspects in everyday encounters based on affection and empathic concern (Kleinrichert, 2024). One step further, scholars in Science and Technology Studies and philosophy have pointed out that concepts such as “objectivity,” “rationality,” “truth,” and “bias” are always anchored in the social relations of a community, notions of well-being, and the particularities of the socio-historical context, criticizing dualistic styles of thinking (Taylor et al., 2023; Zajko, 2021). Hence, experiential mind enables empathic understanding, giving rise to moral feelings of compassion, sympathy, solidarity, or righteous anger, grounding moral judgments (Bierhoff, 2002). On this account, empathic understanding is essential for justice (Tronto, 2020). Empirical research on the perception of AI confirms this view. Compared to humans, AI is considered less permissible for making parole decisions (Bigman and Gray, 2018), less capable of committing moral violations (Shank and DeSanti, 2018), and having a virtuous character to a lower extent (Gamez et al., 2020; Shank et al., 2021) because of the perceived lack of mind in AI, with experiential mind explaining the bulk of the difference.

As a second argument for grounding permissibility in empathic understanding, the ability to share the feelings and motivations of the involved individuals enables a more complete view of the situational circumstances of a particular case (Machura, 2017). Empathy enables a kind of sensitivity towards an individuals’ goals, needs, hopes, desires, and so on, moving beyond and below the application of universal principles (Kleinrichert, 2024; Nallur and Finlay, 2023). Referring again to the ethics of care, when forming judgments, we have a responsibility to pay attention to the details of people’s lives, to hear the full story, and to consider the particular context (Jesenková, 2022; Tronto, 1998, 2020). In the juridical system, understanding the motivations behind an offender’s actions or the victims’ feelings are important pieces of evidence (Xu et al., 2022; Završnik, 2021). Empathic understanding allows agents to take additional information into account and acknowledge the uniqueness of individual cases (Geser, 1989; Nagtegaal, 2021). Again, empirical research supports this argument, showing that the perception of an algorithm as being unable to identify unique characteristics explains the aversion against using AI in personal recruiting (Lavanchy et al., 2023).

Finally, an agent’s ability for empathic understanding might also count as a precondition for successful human interaction. Especially in high-stakes situations (Chan and Lo, 2025), people prefer to interact with an agent that is able to fully understand, react and respond to their needs and worries. Consistent with this idea, Schenk et al. (2024) found that AI is considered much less permissible in the context of cancer diagnosis compared to more repetitive and low-stake tasks, such as fact checking in a newspaper.

### 2.3 Mechanical objectivity vs. empathic understanding

The concepts of mechanical objectivity and empathic understanding represent ideal types (Weber, 2019), summarized in Table 1. Rarely, they are as explicit, pure, and analytically distinct as they are here. Of course, an agent, process, or outcome might incorporate both, striking a balance between the two. They imply various tradeoffs, being polar opposites in many ways. While mechanical objectivity rests on the impersonal application of rules, empathic understanding refers to the ability to share emotional and motivational states of other individuals. Mechanical objectivity has a strong elective affinity to algorithms and machines, empathic understanding is linked to essentially human qualities. While the former is best realized in the absence of mind, the latter presupposes experiential mind. Mechanical objectivity derives permissibility from standardization and consistency, empathic understanding derives permissibility from the comprehensive understanding of unique circumstances. Finally, while mechanical objectivity values rationality, self-discipline, and control, empathic understanding stresses emotions, personal experience, and intuition. From the perspective of mechanical objectivity, a process solely relying on empathic understanding is prone to arbitrariness and special treatment. From the perspective of empathic understanding, a purely mechanically objective process is inflexible, deterministic, and heartless.

**Table 1:** Schematic depiction of the concepts of mechanical objectivity and empathic understanding.

	Mechanical objectivity	Empathic understanding
Foundations	Impersonal application of rules	Sharing of emotional and motivational states
	Absence of mind and freedom from will	Experiential mind
Elective affinity	Machines	Human Beings
Legitimacy	Consistency and elimination of arbitrariness	Comprehensive understanding of unique circumstances
Moral virtues	Self-discipline, rationality, and control	Emotions, personal experience, and uncodifiable human intuition

Whether mechanical objectivity or empathic understanding increase or decrease the permissibility of AI is thus ultimately an empirical question. Still, given that mechanical objectivity is a major justification for using AI in modern societies (Burrell and Fourcade, 2021; Figueroa-Armijos et al., 2023; Lavanchy et al., 2023; Sartori and Bocca, 2023; Xu et al., 2022), we might expect a positive impact on permissibility. Similarly, a lack of empathic understanding might be a major barrier to use AI (Bigman and Gray, 2018; Shank and DeSanti, 2018; Shank et al., 2021). The following two hypotheses follow:

*H1:* The capacity of AI to produce a mechanically objective outcome increases the permissibility of using AI.

*H2:* The capacity of AI for empathic understanding increases the permissibility of using AI.

### 2.4 Social and technological orientations

Given these tradeoffs, the importance of mechanical objectivity and empathic understanding might vary between individuals. However, there is barely any research looking into moderators for the impact of ethical concepts. An exception would be Kieslich et al. (2022) who found several clusters of respondents differing in the relative importance of criteria for ethically designed AI systems, correlating with age, education, and attitudes towards AI. I argue that the importance of mechanical objectivity and empathic understanding varies in theoretically predictable ways depending on general orientations towards technology and sociality. Orientations are internalized dispositions used to perceive and hierarchize external cues (Hashinaga et al., 2023; Schwartz, 2007; Weingartner et al., 2022).

Technological orientation refers to the need to interact with technological artifacts and the value of making new experiences with emerging technologies (Horstmann and Krämer, 2019). Technologically oriented individuals should feel more comfortable with using new technologies to solve social problems (O’Shaughnessy et al., 2023). Research has mostly shown that technological affinity or efficacy, innovation appreciation, prior experience with, and knowledge of AI all contribute to positive attitudes towards AI (Horstmann and Krämer, 2019; Korn et al., 2021; Mantello et al., 2023; Mays et al., 2022; Shulner-Tal et al., 2023; as an exception Yigitcanlar et al., 2022). Some studies hint at an interaction effect between technological orientation and mechanical objectivity. As Graham (2022) has shown in a discourse analysis, academics in technological disciplines emphasize rule-following for the programming of ethical AI more strongly than scholars from humanities, the latter being skeptical of the codifiability of morality. Additionally, in a study by Shin (2022), algorithmic literacy increased the effect of trust on the credibility of algorithmic outputs. Given the elective affinity of mechanical objectivity with technological artifacts (Beer, 2016; Daston and Galison, 1992; Xu et al., 2022), the permissibility of mechanically objective AI should hence increase with technological orientation:

*H3:* The effect of mechanical objectivity on the permissibility of AI increases with an individual’s technological orientation.

Social orientation refers to a need for social interaction and social solidarity (Hashinaga et al., 2023; Ito, 1994). While no study has investigated social orientation, there is research on related constructs. First, most studies analyzing the personality traits of the Five-Factor model found that agreeableness, describing a friendly and helpful personality, leads to favorable attitudes and trust towards AI (Chien et al., 2016; Richert et al., 2018; Shulner-Tal et al., 2023; Stein et al., 2024). However, this personality trait also encompasses being uncritical and optimistic, which is not necessarily the case for socially oriented individuals. Second, research has found that individualism is positively correlated with trust in automation and perceived benefits of predictive policing (Chien et al., 2016; O’Shaughnessy et al., 2023). Since individualists strive for independence from others (Hofstede and McCrae, 2004), which is opposite to social orientation, these results would imply a negative relation between social orientation and the permissibility of AI. Third, it stands to reason that socially oriented individuals might be more averse towards using AI because of its risk to deepen social inequalities, the exclusion of marginalized groups, and the violation of basic human rights (Chan and Lo, 2025). No research so far has investigated a moderating role of social orientation. Since socially oriented individuals value personal connections with other people and their well-being, they should consider empathy, emotions, individual circumstances, and the ability for successful human interaction more important (Hashinaga et al., 2023; Kleinrichert, 2024). I hence expect:

*H4:* The effect of empathic understanding on the permissibility of AI increases with an individual’s social orientation.

## 3 Data and methods

To test these hypotheses, I use data from a survey experiment conducted with university students in Switzerland. Although familiar with the concept of AI, the Swiss population has had limited experience with this technology at the time of the study (just 37% of the population had used such systems in 2023; Latzer and Festic, 2024). They have also been less exposed to human-robot interactions (Statista, 2024) and hold more ambivalent attitudes towards intelligent technologies (Dang and Liu, 2021)—especially compared to East-Asian countries. Turning to the legal system, Swiss people place considerably more trust in courts and the judicial system than the OECD average (69% vs. 54% with high or moderately high trust, OECD, 2024). This is in line with Switzerland being a highly individualistic culture with a stronger orientation towards abstract formal rights and obligations (in contrast to personal ties emphasized in collectivistic cultures; Hashinaga et al., 2023; Hofstede and McCrae, 2004). Assuming that mechanical objectivity is more important in individualistic countries with high trust in the legal system and that empathic understanding is more important when AI/robot-human interactions are uncommon, the tension between empathic understanding and mechanical objectivity could surface in a particularly pronounced way in the Swiss case.

To understand AI’s current and future implementation and regulation, research with university students is highly informative. As studies have shown, individuals with higher education are statistically more likely to attain positions of power, to become entrepreneurs, to vote, and to engage in forms of social activism (Dahlum and Wig, 2021; Verba et al., 1995). Students are hence a highly relevant population to explain the perceived permissibility of AI. Having said this, survey experiments do not presuppose representative samples of the general population, especially if strong interaction effects of the independent variables and sample characteristics (such as education or age) are unlikely (Auspurg and Hinz, 2015).

The survey was conducted at the universities of Zurich and Lucerne. These universities were purposefully chosen to increase the heterogeneity of the sample in terms of the students’ social characteristics. The former is located in the largest city of Switzerland with an urban population. The latter is situated in a smaller city and rural surrounding area with a more conservative population. In Zurich, the survey was distributed via email invitation to students from all faculties in autumn 2021. Participation was incentivized with a lottery. Since an invitation by email was not possible in Lucerne for legal reasons, it was administered in classrooms in autumn 2021, 2022, and 2023. To keep the conditions as identical as possible across research sites, instructors in Lucerne ensured that students filled out the survey on their own. Participation was voluntary in all instances. Respondents gave informed consent at the beginning of the survey. To control for methodological differences between locations or systematic sampling bias, all upcoming regression models include dummy terms for the waves of data collection.^1^

The questionnaire was available in German and English to accommodate the multilingual structure of Switzerland. In total, 793 people participated. A description of the sample shows that 65% of the respondents are enrolled in disciplines from the humanities or social sciences (including psychology, economics, and education) and 35% are enrolled in other fields such as engineering or medicine. The average study length was 4.5 semesters. Female respondents are overrepresented (female = 61%, male = 37%, other = 2%). The mean annual net income is low with 13′000 Francs, typical for a student population. The majority of responses were collected at the University of Zurich with 75% and 25% at the University of Lucerne, reflecting the unequal sizes of these universities.

**Figure 1:**
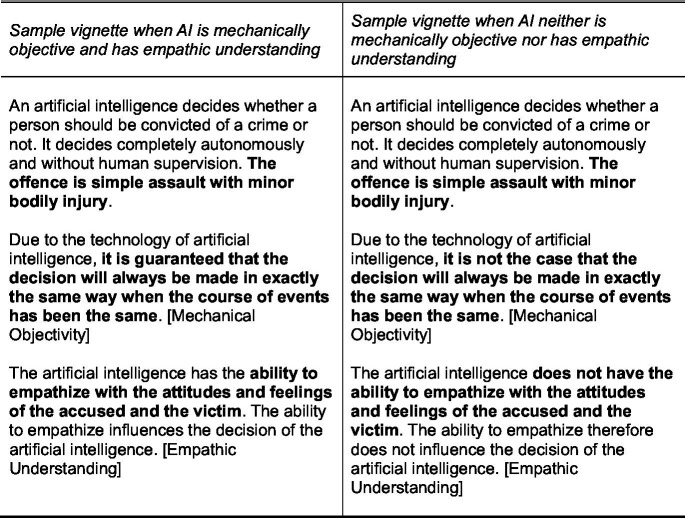
Sample vignettes. Dimensions in brackets. English translation.

The vignettes in the survey experiment describe how an artificial intelligence decides whether a person should be convicted of a crime or not (cf. Bigman and Gray, 2018; Lima et al., 2021; Shank and DeSanti, 2018). I used a 2×2 design with two conditions for mechanical objectivity (yes/no) and two conditions for empathic understanding (yes/no). Figure 1 presents sample vignettes. To manipulate mechanical objectivity, one experimental dimension stated whether the decision made by AI is always the same when the course of events has been the same (or not). Thus, the manipulation directly refers to the most important features of mechanical objectivity for judgments of permissibility, namely that the system always produces the same output with the same input, ensuring standardization, maximizing consistency, and minimizing arbitrariness. To manipulate empathic understanding, the second experimental dimension stated whether the AI has the ability to emphasize with the accused and the victim, influencing its decision (or not). Thus, the manipulation directly refers to the capability of understanding the motivations and feelings of the individuals involved in the crime (for a similar manipulation see Lee et al., 2021). In contrast to some previous studies on the moral permissibility of AI (Bigman and Gray, 2018; Shank and DeSanti, 2018), the experimental design has the advantage of manipulating mechanical objectivity and empathic understanding directly instead of measuring attributions of experiential mind or objectivity ex-post. Experimentally manipulating these factors strengthens the causal interpretation of the statistical effects.

The vignettes held the type of crime constant. The offence is of intermediate severity, namely assault with minor bodily injury. The literature is unclear whether the severity of the crime is relevant for moral judgments. Hong and Williams (2019), for example, found no effects of the severity of a crime on attributions of responsibility to AI. The vignettes represent a futuristic scenario to study the effects of mechanical objectivity and empathic understanding in a stylized setting. First, the vignette specified that AI decides autonomously and without human intervention. To date, AI as a fully independent adjudicating entity has not been implemented in the judiciary, although there have been ambitions to employ fully autonomous AI in courts (Soukupová, 2021; Završnik, 2021). In any case, Lima et al. (2021) only found minor differences in the explanatory factors for the attribution of responsibility between a fully autonomous AI and AI as assisting tool for bail decisions. Second, there is wide agreement that AI does not show signs of experiential mind so far (Harris and Anthis, 2021; Lavanchy et al., 2023). Of course, AI could be programmed to simply imitate emotional capacities, e.g., by using emotive language or emotional cues (Lee et al., 2021; Richert et al., 2018).

At the end of each vignette, respondents rated the permissibility of using AI to decide on the conviction of the accused. Ratings were measured with two items on five-point scales, the second item being reverse coded (see Table 2 for the item wordings, cf. Bigman and Gray, 2018). These items were combined to an index by taking their mean, with higher values indicating stronger permissibility. The reliability is very good with a Cronbach’s alpha of 0.78. I used a within-subject design to administer the vignettes. Respondents rated all four possible combinations of the experimental conditions. The order of the vignettes was randomized. The 2022 wave in Lucerne is an exception, where I introduced a method split using a between-subject design. In total, data collection results in 3007 vignette judgments.

**Table 2:** Wording of the items for permissibility, social orientation, and technological orientation.

	Items wording	Factor loadings		α
		Factor 1	Factor 2	
Permissibility	I think it is right that the artificial intelligence decides on the conviction of the accused	–	–	0.78
	The artificial intelligence should not be allowed to make decisions about the conviction [reverse coding]	–	–	
Social orientation	I value connections with other people	0.67		0.70
	I think it’s important to maintain good relations with the people I interact with	0.56		
	I try to act with integrity toward others	0.55		
	I want to be somebody who helps society (or the people I interact with)	0.57		
	Please rate yourself on the following personal characteristic: being kind	0.50		
Technological orientation	I am interested in new technologies		0.74	0.72
	I am very curious about new technical developments		0.80	
	I know a lot about the topic of artificial intelligence		0.68	
	I have gained personal experience with artificial intelligence or human-like robots		0.43	

English translation. Additionally, factor scores from an exploratory factor analysis of social and technological orientations (varimax rotation). Only loadings above 0.3 are shown. The covariance matrix was computed using multiple imputation for handling missing values. *n* = 793. α = Cronbach’s Alpha.

Turning to the remaining independent variables, social orientation was measured with five items derived from previous research on social personalities (Hofstede and McCrae, 2004; Ito, 1994). In line with the theoretical discussion, these items refer to values of social interaction and social solidarity. A sample item is: “I value connections with other people” (see Table 2 for the wording of all items). Technological orientation was measured with four items (cf. Horstmann and Krämer, 2019). They depict the value of interacting with new technologies, such as artificial intelligence. A sample item is: “I am interested in new technologies.” I computed mean indices for each orientation with a range from one to five. Higher values indicate a stronger social or technological orientation. Reliabilities are good with a Cronbach’s alpha of 0.70 or above. Additionally, an exploratory factor analysis confirms the discriminant validity of the two scales. All items for the orientations load on their respective factors without any cross loadings (see Table 2).

The data is hierarchical with two levels, respondents and vignette judgments. To account for this structure, I use OLS regressions with robust standard errors. To test hypotheses 3 and 4, I computed models with interaction terms with variables centered at their mean. All models include several controls: gender (male, female, other), field of study (humanities and social sciences, other), number of semesters, income (in 10′000 CHF), vignette position, and dummy terms for each wave of data collection. To handle missing values in the regression analysis, I used multiple imputation with 50 imputations (Craig, 2010). Distributions of the imputed data show no anomalies. Finally, a post-hoc power analysis confirms that the number of vignette judgments is sufficient to detect even very small effects of f^2^ = 0.02 at the 5%-level in all regression models. Calculations were performed using R 4.3.0.

## 4 Results

The first regression model includes the two experimental conditions and the controls (see Model 1 in Table 3). Both, mechanical objectivity and empathic understanding, yield highly significant positive effects on permissibility. Respondents find the use of AI as an adjudicating entity in courts more acceptable if it always produces the same output with the same input, guaranteeing a maximum of consistency in its decisions. This is in line with theories referring to standardization, control, and the application of universal rules for fairness perceptions, legitimacy, and trust in the criminal justice system (Farayola et al., 2023; Luhmann, 1978; Machura, 2017; Thibaut and Walker, 1975; Weber, 2019). Respondents value the computational fairness of AI, feeding into ideals of procedural fairness (Burrell and Fourcade, 2021; Shulner-Tal et al., 2023). Likewise, respondents find the use of AI in courts more acceptable if it has the capability to emphasize with the feelings of the involved parties and understand their motivations. This is in line with psychological theories referring to experiential mind for explaining the moral evaluation of AI (Bigman and Gray, 2018; Gamez et al., 2020; Shank and DeSanti, 2018). Criminal sentences should not be passed by an artificial agent without regard for the persons involved (Weber, 2019). They need to take emotions and motivations into account in order to fully understand the unique circumstances of individual cases (Lavanchy et al., 2023; Machura, 2017; Nagtegaal, 2021). Hypotheses 1 and 2 are hence corroborated by the data.

**Table 3:** OLS regression with permissibility as dependent variable.

	Model 1	Model 2	Model 3	Model 4
Mechanical objectivity	0.26***	0.26***	0.26***	0.26***
	(0.03)	(0.03)	(0.03)	(0.03)
Empathic understanding	0.45***	0.44***	0.44***	0.44***
	(0.04)	(0.04)	(0.04)	(0.04)
Technological orientation		0.11*	0.11*	0.11*
		(0.05)	(0.05)	(0.05)
Social orientation		−0.23***	−0.23***	−0.25***
		(0.05)	(0.05)	(0.05)
Objectivity × Techn. Orient.			0.03	0.03
			(0.05)	(0.05)
Empathy × Soc. Orient.			0.26***	0.26***
			(0.07)	(0.07)
Empathy × Techn. Orient.				0.06
				(0.06)
Objectivity × Soc. Orient.				−0.03
				(0.05)
Male gender^1^	0.07	−0.01	−0.01	−0.01
	(0.06)	(0.07)	(0.07)	(0.07)
Other gender^1^	−0.30	−0.33	−0.33	−0.33
	(0.22)	(0.22)	(0.22)	(0.22)
Income (in 10′000 CHF)	−0.01	−0.02*	−0.02*	−0.02*
	(0.01)	(0.01)	(0.01)	(0.01)
Humanities and social science^2^	−0.09	−0.07	−0.07	−0.07
	(0.07)	(0.07)	(0.07)	(0.07)
Semesters	−0.01	−0.01	−0.01	−0.01
	(0.01)	(0.01)	(0.01)	(0.01)
Vignette order 2nd position^3^	−0.05	−0.05	−0.05	−0.05
	(0.04)	(0.04)	(0.04)	(0.04)
Vignette order 3rd position^3^	−0.05	−0.04	−0.04	−0.04
	(0.04)	(0.04)	(0.04)	(0.04)
Vignette order 4th position^3^	−0.10*	−0.10*	−0.10*	−0.10*
	(0.04)	(0.04)	(0.04)	(0.04)
Lucerne 2022^4^	−0.02	−0.03	−0.03	−0.03
	(0.16)	(0.16)	(0.16)	(0.16)
Lucerne 2023^4^	−0.16	−0.13	−0.13	−0.13
	(0.12)	(0.13)	(0.13)	(0.13)
Zurich 2022^4^	−0.17	−0.16	−0.16	−0.16
	(0.10)	(0.10)	(0.10)	(0.10)
Intercept	2.84***	2.84***	2.85***	2.85***
	(0.18)	(0.18)	(0.18)	(0.18)
R^2^	0.06	0.08	0.08	0.08
n	3,007	3,007	3,007	3,007

Clustered standard errors. Unstandardized coefficients. Standard errors in parentheses. *p < 0.05, **p < 0.01, ***p < 0.001. Reference categories: ^1^female gender, ^2^other disciplines, ^3^vignette order 1st position, ^4^Lucerne 2021.

Yet, a look at the size of the coefficients indicates that empathic understanding yields a stronger effect on permissibility than mechanical objectivity, on average. An inferential test on the equality of coefficients supports this interpretation, showing that the effect sizes are statistically different at the 0.1 percent level. Hence, while both concepts causally impact permissibility, empathic understanding is more consequential overall. This underscores the primary role of the attribution of internal states to an agent, emphasized in psychological accounts (Bigman and Gray, 2018; Gamez et al., 2020; Gray et al., 2007; Shank and DeSanti, 2018; Shank et al., 2021), in contrast to the secondary role of rule-following and social control, emphasized in sociological approaches (Beer, 2016; Brayne and Christin, 2021; Burrell and Fourcade, 2021; Daston and Galison, 1992; Geser, 1989; Porter, 1992).

Model 2 adds technological and social orientations. The main effects for both variables are significant. As could be expected from the theoretical discussion and previous research, technological orientation is positively correlated with the permissibility of using AI (Horstmann and Krämer, 2019; Korn et al., 2021; Mantello et al., 2023; Mays et al., 2022; O’Shaughnessy et al., 2023; Shulner-Tal et al., 2023; Yigitcanlar et al., 2022). In contrast, social orientation is negatively correlated. Respondents valuing social interactions and social solidarity are less accepting of using AI as an adjudicating entity in courts. Interestingly, this stands somewhat in contrast to the findings on agreeableness, describing a friendly and helpful personality, repeatedly found to be positively related to favorable attitudes towards AI (Chien et al., 2016; Richert et al., 2018; Shulner-Tal et al., 2023; Stein et al., 2024). One reason might be that the personality type agreeableness conflates various attributes, namely a prosocial orientation on the one hand and an uncritical and optimistic outlook on the other. The former leads to a more skeptical view of AI.

**Figure 2:**
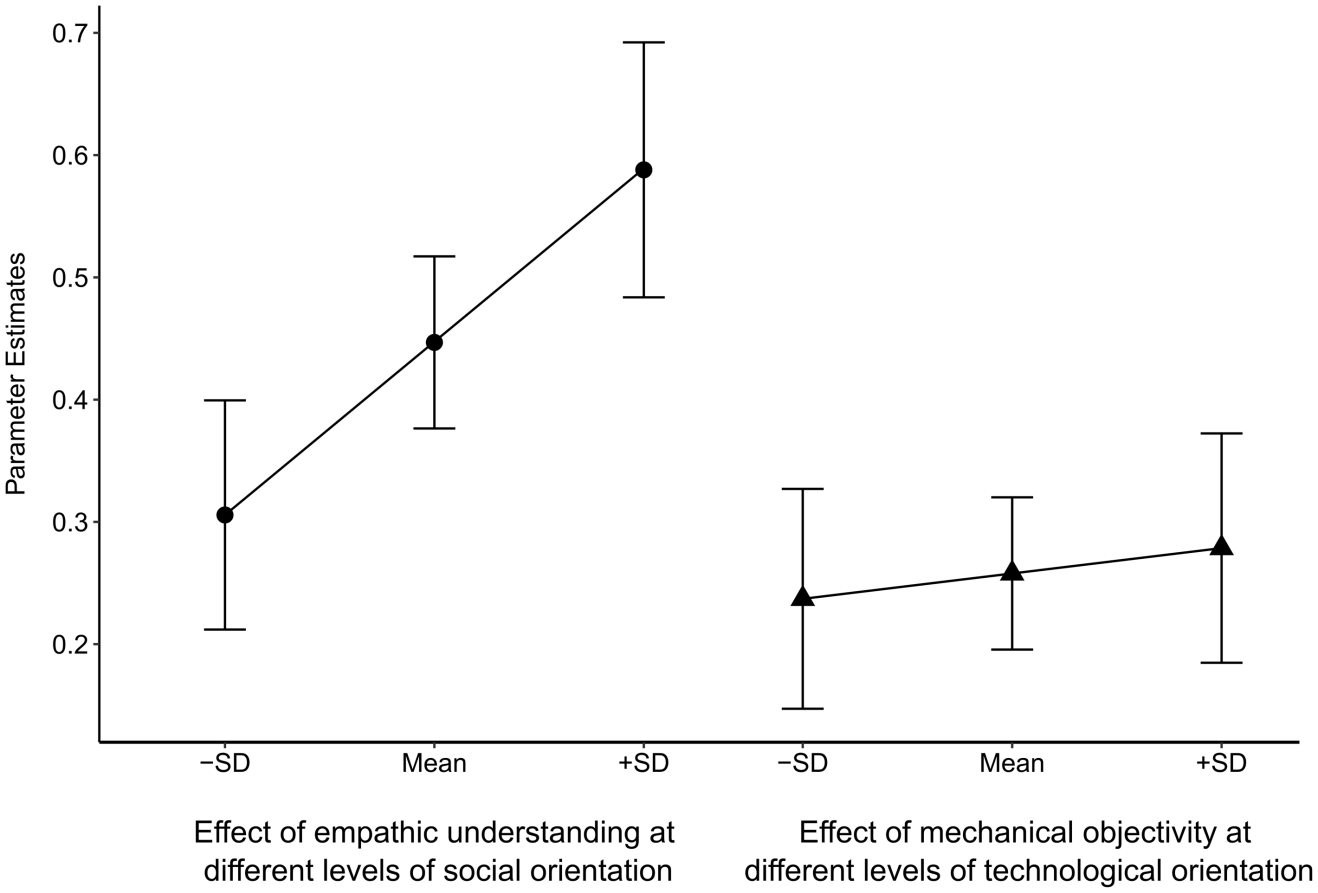
Plots for the interaction effects of empathic understanding and mechanical objectivity with social and technological orientations, respectively. Parameter estimates are unstandardized regression coefficients. SD=Standard deviation.

Model 3 adds the theoretically derived interaction terms between orientations and experimental conditions. In line with hypothesis 4, the interaction term between social orientation and empathic understanding is highly significant. Social orientation moderates the effect of empathic understanding on moral permissibility. Hence, the stronger an individual’s social orientation, the more important the capacity of AI to share the emotional and motivational states of others. Additionally, Figure 2 plots the effects of empathic understanding conditional on social orientation. As we can see, the effect of empathic understanding is nearly twice as large for individuals with a relatively strong social orientation (one standard deviation above the mean) compared to individuals with a relatively low social orientation (one standard deviation below the mean). However, I do not observe a similar moderating effect of technological orientation on the effect of mechanical objectivity. Although the interaction effect is positive, it is statistically insignificant. Figure 2 confirms that the effect size of mechanical objectivity increases marginally with technological orientation. Hypothesis 3 is thus rejected. The effect of mechanical objectivity does not depend on technological orientation, despite various findings hinting at a moderating role (Graham, 2022; Shin, 2022). The final model 4 completes the analysis by adding the remaining possible interaction terms. From a theoretical perspective, no interactions between technological orientation and empathic understanding or between social orientation and mechanical objectivity are to be expected since these orientations are conceptually unrelated to the respective normative frames. In line with this, none of the interaction terms reaches statistical significance.

In total, I thus find clear empirical evidence for individual variation in the relative importance of ethical concepts for the permissibility of AI (cf. Kieslich et al., 2022). Two segments emerge. On the one hand, there are individuals with a weak social orientation. For them, mechanical objectivity and empathic understanding are of similar importance for the permissibility of AI. This is confirmed by a series of tests on the equality of coefficients for mechanical objectivity and empathic understanding at various levels of technological orientation, showing that none of the tests are significant (all *p* > 0.1). On the other hand, individuals with an intermediate or high social orientation consider empathic understanding as more important than mechanical objectivity, irrespective of their technological orientation, as a series of additional tests show (all tests are statistically significant at a 5%-level or less; see also the confidence intervals in Figure 2).

## 5 Conclusion

### 5.1 Mechanical objectivity or empathic understanding?

The conflicting goals of mechanical objectivity and empathic understanding are resurfacing in a particularly clear and powerful way with the accelerated development and continued implementation of artificial intelligence (Clark and Gevorkyan, 2020; Daston and Galison, 1992; Fabian, 2020; Porter, 1992). Mechanical objectivity puts standardization and rules before the particularities of individual cases in order to detach a process from a person’s subjectivity, maximizing consistency. Empathic understanding describes the capacity to share emotional and motivational states of other individuals, enabling a comprehensive interpretation of the unique circumstances of specific cases. While the former is best realized in the absence of mind, showing an elective affinity to algorithms and machines, the latter presupposes experiential mind, showing an elective affinity to being human (Daston and Galison, 1992). There is thus a normative tension. As Kleinrichert (2024) contends, in similar veins to many others, how can AI be technologically neutral but at the same time caring in human interactions?

Based on a large-scale survey experiment with students in Switzerland, I find that both mechanical objectivity and empathic understanding causally impact the permissibility of using AI as an adjudicating entity in courts. Respondents strike a balance between both concepts. Yet, the balance tips according to an individual’s social orientation. The relative importance of moral concepts for permissibility judgements systematically varies between individuals in theoretically predictable ways (cf. Kieslich et al., 2022). Respondents with a weak social orientation find both equally important. For respondents with an intermediate or strong social orientation, empathic understanding is significantly more relevant than mechanical objectivity. Technological orientation, in contrast, does not moderate the relative importance of these ethical ideals. This details the scope conditions for sociological and psychological explanations of the permissibility of AI (Bigman and Gray, 2018; Brayne and Christin, 2021; Burrell and Fourcade, 2021; Gamez et al., 2020; Geser, 1989; Shank et al., 2021). They have similar explanatory power in a population with weak social orientations. In contrast, psychological accounts referring to the attribution of mental states turn out to be more valuable for explaining the permissibility of AI in a population with socially oriented individuals.

There is thus a certain mismatch between the public discourse of AI advocates and the perception of non-experts. While the former often praise this new technology for making a process more objective, consistent, and accountable (Brayne and Christin, 2021; Burrell and Fourcade, 2021; Geser, 1989; Lavanchy et al., 2023; Petre, 2018; Rosen et al., 2021; Sartori and Bocca, 2023; Zajko, 2021), the latter find an agent’s capacity to share the emotional and motivational states of others at least as important, if not more important. Actually, we could expect mechanical objectivity to play a larger role in modern, highly rationalized societies, especially in relation to new technologies (Burrell and Fourcade, 2021; Nagtegaal, 2021; Weber, 2019; Xu et al., 2022). Mechanical objectivity empowers weaker individuals by ensuring standardized procedures (Porter, 1992), something that current systems of AI may actually accomplish, also in the legal domain (Farayola et al., 2023; Stevenson and Doleac, 2019; Xu et al., 2022). In contrast, current AI lacks the capacity for empathic understanding. Perhaps it is exactly this lack of emotion and experiential mind making it so important to non-experts for their permissibility judgments.

I hence find a clear “empathy gap” for AI’s permissibility—an important insight for normative discussions on AI, which have predominantly focused on privacy, transparency, bias, or social exclusion. While undoubtedly of crucial importance (Chan and Lo, 2025; Kieslich et al., 2022; Schenk et al., 2024), these issues should be discussed in conjunction with the normative demand for empathic understanding, especially in high-risk domains, such as the legal system (European Union, 2024; Kleinrichert, 2024; Nallur and Finlay, 2023; Tronto, 2020).

### 5.2 Mechanical objectivity and empathic understanding in practice

From a practical perspective, this empathy gap might be addressed in three different ways. First, if we assume that achieving empathy is impossible for AI, at least for the time being, we can follow Xu et al. (2022) concluding that an empathy gap can only be filled by human beings, thus confining AI to certain assisting tasks and collaborations with human agents. Having a human in the loop, be it in a supervising position, is clearly beneficial for trust in AI, representing an additional safeguard against the unfair treatment of individual cases (Fabian, 2020). It also comes at unexpected costs. When working in teams, individuals have used AI as scapegoats, shifting moral blame to the technological system and blurring accountabilities (Kneer and Christen, 2024; cf. Lima et al., 2021). Some tentative research in medical diagnostics also shows that overall performance might be lower in hybrid human-AI systems compared to AI alone (Goh et al., 2024). While delegating AI to a merely assistive function might seem like a simple solution at first glance, it opens up a completely new can of worms.

Second, one might try to mimic empathic capacities by implementing social and emotional cues in AI (Lee et al., 2021; Richert et al., 2018). While research is inconclusive whether anthropomorphic cues are actually sufficient to change behavioral responses (Bonnefon et al., 2024; Schenk et al., 2024), this raises normative questions, too. Some scholars have argued for leveraging anthropomorphic tendencies in the domain of law (e.g., Darling, 2015). Yet, such approaches have been accused of humanwashing more recently (Scorici et al., 2024). Strategically exploiting the tendency to anthropomorphize machines has been called “deceptive” or “manipulative.” The AI Act by the European Union clearly prohibits deceptive manipulation in the deployment of AI (European Union, 2024). In any case, the non-intended consequences of humanwashing might be even more far-reaching. As we have seen in the present analysis, increasing the saliency of empathic understanding by emotional cues also draws away attention from the risks of the conservative tendencies inherent to a mechanically objective AI (Zajko, 2021).

Third, the empathy gap can also underscore Nallur and Finlay’s (2023) more recent call to intensify the engineering of empathic capabilities in AI, comprising emotion recognition, understanding, or even affective concern (Kleinrichert, 2024; Mantello et al., 2023). Yet, it is highly controversial whether true empathic understanding can ever be achieved for AI, let alone in the near future (Harris and Anthis, 2021). Even if we are optimistic about the possibility of empathic AI, this strategy is feasible in the mid or long run at best. In the meantime, various stakeholders, from professionals (e.g., judges and lawyers) to citizens (e.g., legal subjects), should be involved in the creation of safeguards and regulations, tackling unprecedented challenges of AI, including blurred responsibilities in human-AI-cooperation or humanwashing (cf. Chan and Lo, 2025).

Independent of these strategies, given we want to increase the acceptance of AI, we should focus on the concerns of socially oriented individuals. These individuals are especially skeptical. Not only does a social orientation result in an overall lower permissibility of using AI in the first place. AI’s deficit in empathic understanding weighs particularly heavy for socially oriented individuals. Hence, focusing on the concerns of socially oriented individuals might yield a double dividend. Not only do such strategies have a general effect on overall acceptance, targeting the worries of socially oriented individuals regarding an AI’s empathy gap would be especially efficient. On the flipside, the public should be made aware of the pitfalls of mechanical objectivity. This is especially relevant for a population segment with a less pronounced social orientation, for which mechanical objectivity is more consequential. Mechanical objectivity should be recognized as a distinct concept in critical AI discourse (Zajko, 2021).

### 5.3 What’s next for mechanical objectivity and empathic understanding?

I want to point out three limitations and avenues for future research. First, students are a highly relevant population to study the reception and implementation of AI among non-experts. In contemporary western societies, educated individuals are statistically more likely to attain higher social positions in various fields and to be politically active (Dahlum and Wig, 2021; Verba et al., 1995). This is not to say, however, that other social groups are powerless in the social struggle around the legitimate implementation of AI. AI might even provide new resources to dominated groups to defend their interests (for the legal domain see Soukupová, 2021). Future studies should collect data from the general population to increase population validity and to arrive at a more fine-grained picture of the social divides in the moral perception of AI.

Second, as an individualized country with limited exposure to AI in the years of the study, Switzerland provides a particular cultural, legal and technological context. The extent to which the present results can be generalized to other countries is an open question. One might theorize that empathic understanding becomes more important in collectivistic cultures, emphasizing personal connections, while it might become less important with advanced exposure to AI (Hofstede and McCrae, 2004). There is a dire need for systematic comparative research testing such hypotheses on cross-country differences.

Finally, a stronger focus on mechanical objectivity and empathic understanding was warranted given the scope of the study, juxtaposing two logically opposing ideals. However, the experimental design could be extended in various directions. It would be highly valuable, for example, to compare the effects of mechanical objectivity and empathic understanding between AI and human agents, to compare the effects of mechanical objectivity to bias or reliability, or to compare various social domains. The latter could be most promising: Recent research has repeatedly shown how moral judgments of AI and related technologies differ between situational contexts (Gerdon, 2024; Schenk et al., 2024). One should try to explain this situational variation, for example by comparing high- vs. low-stakes situations (e.g., the severity of the crime), task repetitiveness (e.g., various types of contract law), or societal systems (commercial and criminal law). We lack theoretical models accounting for such situational effects.

The reception of new technologies needs to be explained as a socially embedded phenomenon. Concepts such as “mechanical objectivity,” “empathic understanding,” “algorithm,” or “AI” have developed historically (Beer, 2016; Daston and Galison, 1992), they are contested in social fields (Sartori and Bocca, 2023), and are part of a culturally shared stock of knowledge (Abend, 2014). Individuals put these concepts to work for the categorization of new technologies, giving meaning to concrete situations. They apply “machine heuristics” in encounters with AI, evoking notions of objectivity, efficiency, empathic concern, heart, and so on, shaping expectations, evaluations, and behaviors (Mays et al., 2022).

Yet, the relative importance of such cultural concepts varies in theoretically predictable ways between individuals based on their internalized orientations. General orientations are used to rate and hierarchize external stimuli (Weingartner et al., 2022). Social orientation has received little to no attention in research on the perception of AI. Social orientation is a distinct theoretical concept, not to be confused with other personality traits (i.e., agreeableness), which should be researched thoroughly. Personal orientations, values, traits, and beliefs are crucial for explaining the reception of AI, apart from situational circumstances (Mays et al., 2022). Thus, only by theorizing the interplay between historically formed concepts and socially differentiated orientations, we are able to explain the moral permissibility of AI as a socially embedded phenomenon.

## Data Availability

The raw data supporting the conclusions of this article will be made available by the authors, without undue reservation.

## Footnotes

^1^None of them reaches a conventional level of statistical significance, however. Furthermore, I computed a regression model with all possible interactions between theoretically relevant variables (mechanical objectivity, empathic understanding, social orientation, technological orientation, and all of their interaction terms) and a dummy term for the locations (Zurich vs. Lucerne). None of these additional interactions were statistically significant at the 5%-level or less and all of the findings are entirely robust. Hence, there is no indication that effect sizes significantly differ between locations.
